# Tumor cells and their crosstalk with endothelial cells in 3D spheroids

**DOI:** 10.1038/s41598-017-10699-y

**Published:** 2017-09-05

**Authors:** Hila Shoval, Adi Karsch-Bluman, Yifat Brill-Karniely, Tal Stern, Gideon Zamir, Ayala Hubert, Ofra Benny

**Affiliations:** 1 The Institute for Drug Research, The School of Pharmacy, Faculty of Medicine, The Hebrew University, Jerusalem, Israel; 2 Department of Surgery and Transplantation Unit, Hadassah Medical Center, Ein-Karem, Jerusalem Israel; 3 Oncology Department- Hadassah Medical Center, Ein-Karem, Jerusalem Israel

## Abstract

Recapitulating the tumor microenvironment is a central challenge in the development of experimental model for cancer. To provide a reliable tool for drug development and for personalized cancer therapy, it is critical to maintain key features that  exist in the original tumor. Along with this effort, 3-dimentional (3D) cellular models are being extensively studied. Spheroids are self-assembled cell aggregates that possess many important components of the physiological spatial growth and cell-cell interactions. In this study we aimed to investigate the interconnection between tumor and endothelial cells (EC) in hybrid spheroids containing either tumor cell (TC) lines or patient derived cancer cells. Preparation protocols of hybrid spheroids were optimized and their morphology and tissue-like features were analyzed. Our finding show that capillary-like structures are formed upon assembly and growth of TC:EC spheroids and that spheroids’ shape and surface texture may be an indication of spatial invasiveness of cells in the extra-cellular matrix (ECM). Establishing a model of hybrid tumor/stroma spheroids has a crucial importance in the experimental approach for personalized medicine, and may offer a reliable and low-cost method for the goal of predicting drug effects.

## Introduction

Personalized cancer therapy is increasingly recognized as the next generation of therapeutic approaches. It is well established that tumors display substantial heterogeneity in their type, site and stage. Even patients with the same type of disease may present quite different tumors’ phenotype^1, 2^. In order to choose an efficient therapy, one must deal with the vast complexity of tumor biology. Several approaches are currently being developed for personalized therapy, including *in silico* prediction tools^3^, genetic analysis^2, 4^ and experimental models^5^. For instance, extraction of genetic information by deep sequencing techniques enables identification of mutations in oncogenes, which can direct clinicians towards certain courses of treatment^6^. However, most current genetic information is poorly translated into clinical treatment plans as a result of a lack of specific key gene targeted drugs. Additionally, the massive tumor heterogeneity often challenges the tumor representation mapping post biopsy, since there is great diversity in the genetic information obtained from different biopsies of the same tumor^7^. Due to these major complexities of cancer, there is currently a great need to develop predictive drug performance tools with clinical relevance. Therefore, reliable experimental models that would predict the overall cell functionality in a physiologically relevant manner, are of high value.

Cellular monolayer assays are commonly used as research tools for drug screening, and are widely applied in molecular biology for the identification of different molecular pathways, among other utilizations^8, 9^. Drug screening of compound libraries for various activities, such as anti-cancer activity, relies mainly on cytotoxicity assays, using established cancer cell lines grown in 2-dimentional (2D) cultures that exhibit rapid growth kinetics. This approach has contributed significantly to an understanding of tumor biology and to anticancer drug discovery and development. However, 2D cell cultures lack key features that are critical for recapitulating physiological systems^10^, such as spatial cell-cell interactions, extra-cellular matrix (ECM)^11^, dynamic metabolic demand and increased hypoxia due to mass growth^12^, and effects of tumor microenvironment^13^. These differences account for the distinct rate of proliferation and cell susceptibility to death signals in the 3-dimentional (3D) model compared with 2D cultures, in response to drug exposure. Previous publications showed reduced cell proliferation rate when cancer cells were grown in 3D cultures compared with the 2D format^14, 15^. The low level of physiological relevance of 2D cultures in cytotoxicity assays sometimes leads to misinterpretation and poor prediction of *in vivo* behavior. These limitations of drug screening in monolayer models may partly be responsible for the high rate of clinical trial failures of new molecules, despite their excellent antitumor properties *in silico* and *in vitro* ^16^.

To that end, 3D cellular models are being developed and studied extensively^17^. One of the most interesting 3D cellular models is the multicellular tumor spheroid model. Spheroids are self-assembled cell aggregates that have the capacity to possess many important components of the physiological spatial growth and cell-cell interactions^18^. These 3D entities create *ex vivo* micro-tissues with metabolic activity that is governed by nutrient and oxygen diffusion mechanisms^18, 19^ similar to avascular tumors. Spheroid diffusion is limited to only 150–200 µm^20^; in larger spheroids, which can reach up to 400–500 µm in diameter, the outer layer continues to proliferate while the core becomes necrotic due to hypoxia and nutrient deficiency. These conditions are similar to hypoxic micro-tumors *in vivo* that are known to negatively affect the sensitivity of the tumor to anti-cancer drugs, and contribute to the acquired resistance^21, 22^.

To better imitate the tumors’ microenvironment *in vivo*, tumor cells (TC) should be grown in the presence of stromal cells that also exist in the tumor niche. Crosstalk between TC and endothelial cells (EC) governs the critical process of the formation of new blood vessels^23^, known as angiogenesis, which is a hallmark of tumorigenesis. The complex interconnection between TC and EC contributes to the modifications in the gene expression profile of EC^24^ and their activation state, which initiate angiogenesis and contribute to drug resistance^25, 26^. A recent study shows a more complex stroma-mimicking spheroid model in which, in addition to TC and EC, mesenchymal cells (MSC) were also included in the co-culture to better sustain and recapitulate the microenvironment, suggesting an enhanced platform for studying the effect of drugs *in vitro* ^11^. Indeed, as a critical component of the tumor tissue, the endothelium should be an integral part of *ex vivo* models. Therefore, great effort is expended to develop spheroids of tumors with endothelial cells, mainly as a model for angiogenesis^12, 27–29^, and to construct interacting capillary systems using microfluidic techniques^30–33^. To better understand the cancer cell-specific behaviors in 3D multicellular structures and their interactions with EC, in this study, we investigated the interactions of TC with EC in 3D in different sources of tumor cells and in different ratios of TC:EC. Our results show that we have successfully developed an optimized protocol for spheroid assembly using the spheroid array method, and characterization of spheroids derived from either cancer cell lines or patient cancer cells. For this purpose we used EC, Human Umbilical Vein Endothelial Cells (HUVEC), that are commonly used for modeling angiogenesis in 3D cultures^24, 27, 28, 34–37^. In addition, we detected potential correlations between the spheroids’ shape and surface texture and the spatial invasiveness of cells in ECM. Establishing a model of hybrid tumor/stroma spheroids is of crucial importance to the experimental approach in personalized medicine. It may also offer a reliable and low-cost method to recapitulate the tumor microenvironment for the goal of predicting drug effects.

## Materials and Methods

### Statement

All experiments and methods were performed in accordance with relevant guidelines and regulations. All experimental protocols were approved by a named institutional/licencing committee. Specifically, human cell collection were approved by the Institutional Review Board (IRB)/Ethics (Helsinki) Committee of the Haddasah Medical Center (#920051034, and 0628-14-HMO). Informed consent was obtained from all subjects, and all methods were carried out in accordance with the relevant guidelines and regulations of the approved IRB protocols.

### Cell culture

Human melanoma cell line A375, human pancreatic cell lines BxPC3, PANC1 and human mammary cancer cell line MDA-MB-231 were obtained from ATCC (VA, USA). A detailed table of all the cell types used for this experiment was added to the supplementary section (Table 1S) HER-positive patient-derived mammary tumor cells BR-58, and melanoma cells derived from patient’s lymph node M21, were isolated from fresh cancer patient biopsies and were kindly provided by Prof. Michal Lotem under the required approvals (see statement above). Human umbilical vein endothelial cells (HUVEC) were purchased from Lonza (Switzerland). All cells were maintained in 10% fetal calf serum (FCS) media with Penicillin/Streptomycin and kept in a humidified incubator at 37 °C with 5% CO_2_. For MDA-MB-231, PANC1 and A375 cell lines, DMEM (Life Technologies, MA, USA) was used, and BxPC3 was maintained in RPMI-1640 (Life Technologies, MA, USA). HUVEC cells were grown in a specific medium, supplemented with PeproGrow-MicroVkit (ENDO-BM & GS-MicroV). Both BR-58 and M21 were maintained in a mixture of DMEM, RPMI1640 and F12 (Life Technologies, MA, USA) with 5% HEPES.

### Spheroids formation using multi-well agarose-coated plates

Agarose hydrogel 1.5% (100 µl) was added to each well of a 96-well culture plate (Thermo Fisher Scientific, Denmark), and incubated at 37 °C for 2 hours. A375, BxPC3, PANC1 and MDA-MB-231cells were seeded in 100 µl growth medium at a concentration of 5,000 cells per well. Plates were incubated at 37 °C for an additional 72 hours to allow formation of 3D spheroids in culture.

### Spheroid formation using U-shaped 96-well plates

Round-bottom 96-well plates (Lipidure^®^, NY) coated with 2-methacryloyloxyethyl phosphorylcholine (MPC) were seeded with 5,000 cells per well of A375, BxPC3, PANC1 and MDA-MB-231 cell lines. Plates were incubated at 37 °C for an additional 72 hours to allow for spheroid formation.

### Spheroid formation using the hanging-drop method

Lids of 10 cm culture plates (Corning, NY) were seeded with 30 μl growth media containing 5,000 cells of either A375, BxPC3, PANC1 or MDA-MB-231. To prevent dehydration of the drops, 5 ml PBS were added to the bottom of the plate. Cells were incubated at 37 °C and 5% CO_2_ for 48 hours to allow aggregation. Once cells created aggregates in the drops, spheroids were collected and transferred to a 10 cm culture plate coated with 0.75% agarose in 10 ml growth medium for an additional 5 days.

### Spheroid formation using Petri Dish well array

Master 3D Petri Dish^®^ 35-well array (Microtissues Inc., RI, USA) was used to create 2% agarose hydrogel micro-wells. Micro-wells were then incubated with 1 ml of the appropriate medium for 60 minutes, followed by seeding of different cells. Cells used in this assay were PANC1, BxPC3, A375 and MDA-MB-231, and two patient-derived cells: BR-58 and M21. Each spheroid contained 5,000–25,000 cells. 10 minutes after seeding, 1 ml of medium was added and templates were incubated at 37 °C for 72 hours. For hybrid spheroid experiments, cancer cells were mixed in different ratios with HUVEC, consisting a total of 10,000 cells per spheroid. TC:EC ratios used for this experiments were: 1:0, 2:1, 1:1, 1:3, 1:8 and 0:1. Spheroids were formed using the 3D Petri Dish^®^ templates as previously detailed (over 72 hours). ImageJ (NIH, MD http://imagej.nih.gov/ij/) was used to generate the calculated area of the different spheroids as detailed in the Spheroids image analysis section below.

### Spheroids invasion collagen assay

A375, BxPC3, M21 and BR-58 spheroids were fabricated in a Petri dish array using 3,000 cells per spheroid. Prior to seeding, the cells were stained by Dil (Invitrogen, CA, USA) for 20 minutes and washed twice with PBS. 24 hours post incubation, spheroids were harvested and embedded in a solution of 0.25% methyl cellulose in the appropriate media. Spheroids were then mixed with collagen (Rat tail collagen type I, Corning, NY) in pH7, seeded in 24-well plates and incubated at 37 °C for 30 minutes. After the gel has set and stabilized, in order to ensure proper media supply, spheroids were detached using a tip, and 300 µl of proper media were added per well. Pictures were taken every few hours using an inverted fluorescent microscope (Olympus IX73).

### Spheroids invasion Transwell assay

Spatial cell invasion assay was performed using 96-well cultured plates with 8 μm pore size polycarbonate membrane transwell inserts (NuNC, Denmark). Cells of A375, M21, BR-58 and BxPC3 at a concentration of 5,000 cells per spheroid were seeded in 75 μl of serum free media on an insert that is coated with 50 ng/ml collagen (type I). Lower compartment was added with 250 μl of 10% FCS containing media. Plates were then incubated at 37 °C for 48 hours to allow cells to migrate. Media was removed from the lower compartment and cells were fixed on the lower side of the inserts filter with a 2 minute incubation of 4% paraformaldehyde (PFA), and washed twice with PBS. Cells were then incubated with 80% methanol in DDW for 20 minutes and washed twice with PBS. Cells on the lower side of the filter were stained with Giemsa for 30 minutes at room temperature (RT) protected from light (1:25 Giemsa in PBS). Staining buffer was removed, upper side of the membrane was carefully swabbed and inserts were air-dried. Using a microscope, cells on the lower side of the membrane were counted, three different fields per well, and an average number of migrated cells was calculated.

### Immunofluorescence and immunohistochemical staining

Spheroids were embedded in an optimum cutting temperature compound (OCT), frozen on dry ice, and stored at −80 °C. Frozen sections (10 μm) were cut using Cryostat CM1950 (−20/−19 °C). All staining incubations were performed at RT and all reagents were rinsed once with PBS. Sections were fixed in PFA 4% for 20 minutes and washed 3 times for 5 minutes. Sections were then incubated for 30 minutes in 3% normal goat serum (Vector Laboratories, California) for blocking. Blocking was removed and anti-CD31 (Abcam, MA, USA, 1:50) in 3% normal goat serum was added for an overnight incubation at 4 °C. Slides were washed 3 times for 5 minutes and incubated with a secondary antibody labeled with Alexa fluor 488 (goat anti rabbit, Abcam, MA, USA) (1:100) in 3% normal goat serum for 1 hour. Sections were then washed 3 times before incubation with DAPI. Mounting media was applied on the slides and samples were visualized using a fluorescent microscope (Olympus IX-73). Immunohistochemically staining was performed using DAB kit as detailed in the Supplementary Information.

### Spheroid image analysis

In order to determine the circularity of the spheroids, an image analysis was performed using MATLAB. Border coordinates were set to define the spheroids’ margin, and marked with a yellow circle. Roundness (R) and smoothness (χ) of the spheroids were measured, parameterizing the global and local circularity, respectively.

Discrepancies of each border coordinates set $\{{{\rm{n}}}_{{\rm{i}}}\}$, were defin﻿ed as

$${\rm{\sigma }}(\{{{\rm{n}}}_{{\rm{i}}}\})\equiv \sqrt{\frac{1}{{\rm{N}}\{{{\rm{n}}}_{{\rm{i}}}\}}\sum _{\{{{\rm{n}}}_{{\rm{i}}}\}}{(\frac{{{\rm{r}}}_{{\rm{i}}}}{{{\rm{r}}}_{{\rm{i}}}}-{\rm{s}})}^{2}}$$

where ${{\rm{r}}}_{{\rm{i}}}$ is *i* border pixel coordinate’s distance from the center of the circle, and ${\rm{N}}\{{{\rm{n}}}_{{\rm{i}}}\}$ being the number of bins in the summation. R is defined by ${\rm{\sigma }}(\{{{\rm{n}}}_{{\rm{i}}}\}=\{{{\rm{M}}}_{{\rm{i}}}\})$ where $\{{{\rm{M}}}_{{\rm{i}}}\}$ is composed of all border points. R provides a multi-range measurement of the divergence of the spheroid from circularity, whereas smaller ${\rm{R}}$ represents rounder spheroids.

χ accounts for local dispersity of the border coordinates. Spheroid border was divided into 32 circular sectors, each defined by an angle of π/16. The size of the angular sector was selected based on the relevant length scale of the spheroids roughness *(other values of the angular sector were tested and produced similar results)*. $\{{{\rm{n}}}_{{\rm{i}}}({\rm{\theta }})\}$ is a set of border coordinates within a given sector. Smoothness was defined as the average of local discrepancies over all ${\rm{\theta }}$ sectors: ${\rm{\chi }}={\rm{\sigma }}{(\{{{\rm{n}}}_{{\rm{i}}}({\rm{\theta }})\})}_{{\rm{\theta }}}$. Therefore, the smaller the value of ${\rm{\chi }}$, the smoother the spheroid is.

To parameterize the size of the different cell types’ spheroids, an image analysis was performed using ImageJ software. Average spheroid size was calculated for each cell amount (5,000, 10,000, 20,000 and 25,000) separately. Spheroids’ size was normalized using:

$$\frac{{\bf{s}}{\bf{p}}{\bf{h}}{\bf{e}}{\bf{r}}{\bf{o}}{\bf{i}}{\bf{d}}{\bf{s}}\,{\bf{a}}{\bf{v}}{\bf{e}}{\bf{r}}{\bf{g}}{\bf{e}}\,{\bf{s}}{\bf{i}}{\bf{z}}{\bf{e}}}{{\bf{5000}}\,{\bf{c}}{\bf{e}}{\bf{l}}{\bf{l}}{\bf{s}}\,{\bf{p}}{\bf{e}}{\bf{r}}\,{\bf{s}}{\bf{p}}{\bf{h}}{\bf{e}}{\bf{r}}{\bf{o}}{\bf{i}}{\bf{d}}{\bf{s}}\,{\bf{a}}{\bf{v}}{\bf{e}}{\bf{r}}{\bf{g}}{\bf{e}}\,{\bf{s}}{\bf{i}}{\bf{z}}{\bf{e}}\,}\,\times 100 \% .$$

### Statistics

*In vitro* data is presented as mean ± SD. Significant differences between cells’ invasion of spheroids were assessed using unpaired two-tailed Student’s t-test, and p < 0.05 was considered statistically significant.

## Results

### 3D template array yielded the more uniform spheroids compared with other methods

**Figure 1:**
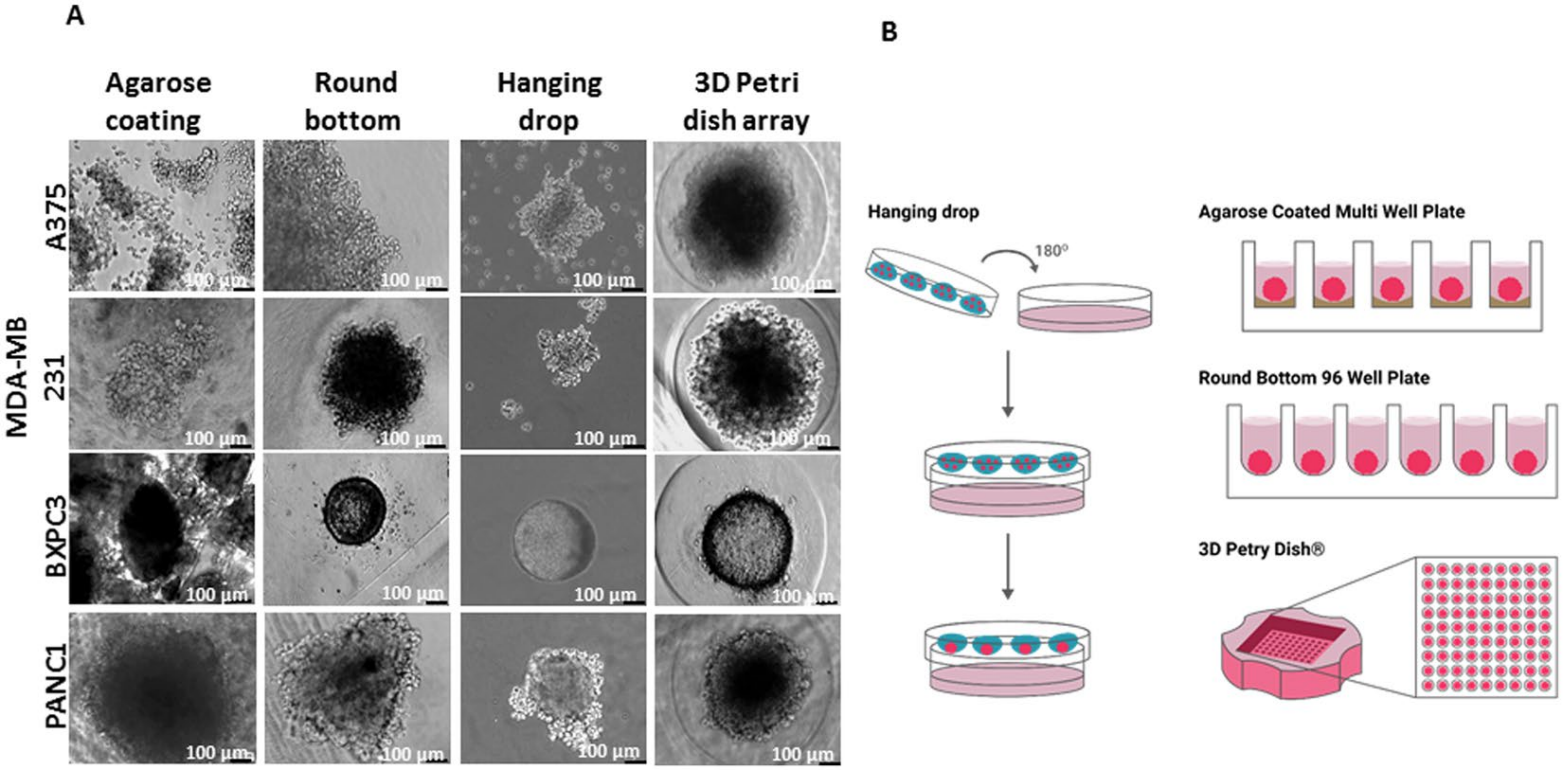
Spheroids growth using different techniques, a comparison between spheroids that were prepared using the following formats: 96 well plate with agarose; Rounded bottom 96 well plate; 3D petri dish with 35 wells; Hanging drop method. Spheroids were made of 5,000 cells per spheroid. Assembly time was 3 days for all methods except the hanging drop method which was 5 days. Bar = 100 µm.

To investigate which method yields the optimal most monodispersed multi-cellular spheroids, we followed the size and shape of four distinct tumor types, using four different methods of preparation. PANC1, BxPC3, A375 and MDA-MB-231 cell lines were used (Table 1S), and applied methods for spheroid formation were: (1) Agarose-coated 96-well plate; (2) MPC-coated U-shape bottom 96-well plate; (3) hanging-drop technique; and (4) 3D template array. All comparisons of spheroids formation are shown in Fig. 1A. Figure 1B portrays a detailed scheme of all the methods used for spheroid fabrication.

Cells grown in agarose-coated 96-well plates were self-arranged in multi cell aggregates to form heterogeneous spheroids according to their shape, size and morphology. Based on the concept of non-adherent coatings, we used Lipidure^®^ MPC-coated 96-well plates to form spheroids (Fig. 1). In this method, all cells tested were self-assembled into a round single spheroid per well. Spheroid’s roundness varied between cell types. For example, PANC1 formed elongated aggregates compared with all other cell types. Notably, spheroid size was cell dependent, and each cell type yielded a different and typical spheroid size. For instance, A375 provided spheroids that were over 10-fold larger than the smallest spheroids formed by BxPC3. In addition to these multi-well methods, spheroids made using the hanging-drop technique were also evaluated. In the hanging-drop method, spheroids were formed only after a relatively long period of 5 days and were of random size and shape, making it impossible to control either one of these parameters. The only cell type that yielded perfectly rounded spheroids was BxPC3, after only 1 day, as seen in Fig. 1A.

A recently developed method of mold array, 5 × 7, enables the imaging of multiple spheroids in a single imaging field^38^. In this method, we used Master 3D Petri Dish^®^ templates made of agarose-coated U-shaped micro-wells. This method yielded uniform spheroids of similar size, with a relatively round and circular shape. While the method produced a monodispersed batch of spheroids in each cell line, the size of spheroids remained relatively unchanged over time (Fig. 1S), compared with the MPC-coated 96-well plate method.

### Effect of cell number on spheroid size and density

**Figure 2:**
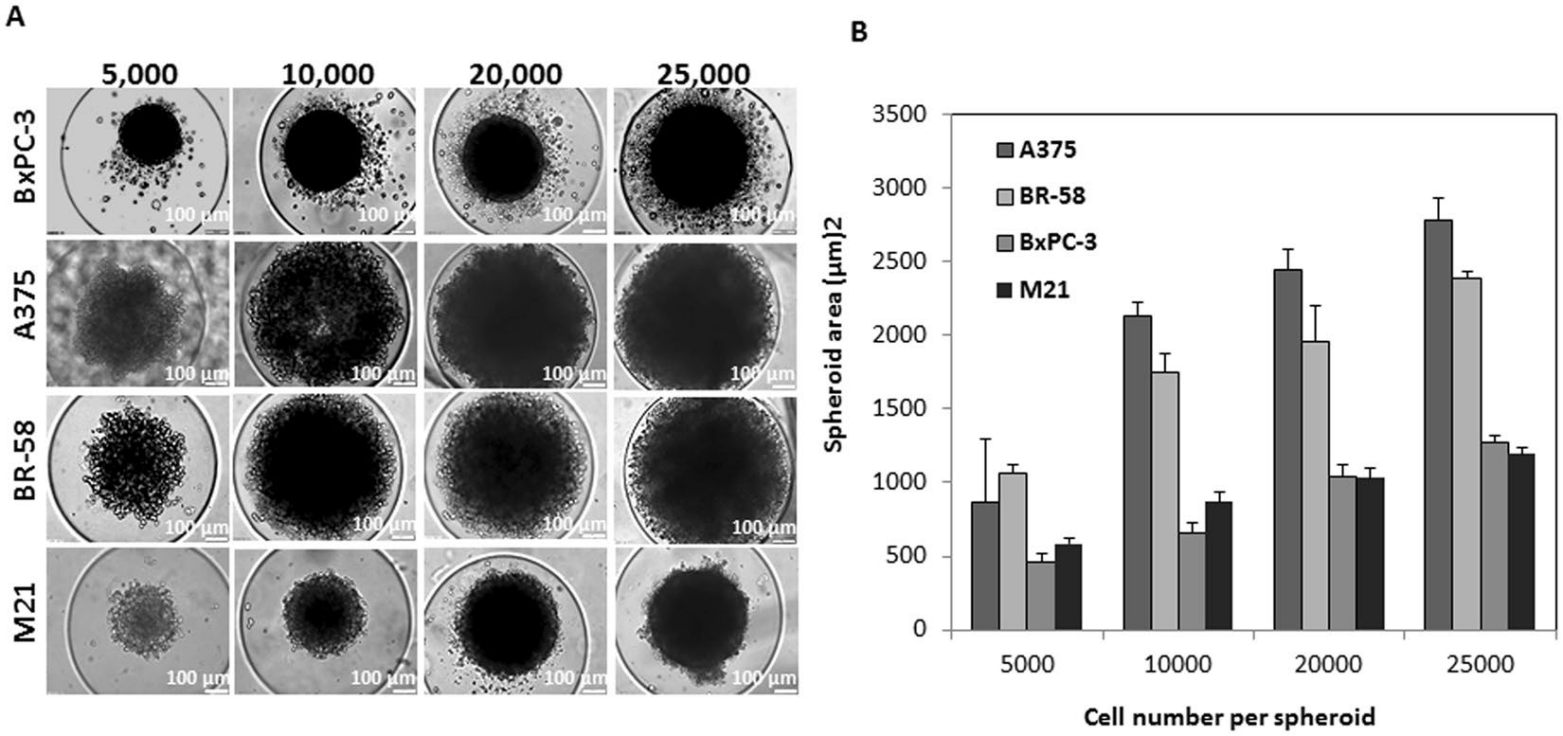
Spheroids made of patient tumor cells or tumor cell lines. **(A)** Typical images of spheroids made of BxPC3, A375, BR-58, and M21. Spheroids were fabricated using different number of cells per spheroid: 5,000, 10,000, 20,000 and 25,000. The spheroids were prepared using the 3D petri dish with 35 well array. **(B)** Graph showing spheroid area values (µm)^2^ as a function of the number of cells per spheroid. n = 4, Bar = 100 µm.

Experiments of spheroid formation with the 3D petri dish template were performed with 5,000, 10,000, 20,000 and 25,000 cells per spheroid, using two patient-derived cell types; M21 and BR-58, and two cancer cell lines: A375 and BxPC3 (Fig. 2A). While all cell lines formed spheroids, increasing the number of cells did not produce a consistent pattern of growth in spheroids’ size. For a simple uniform aggregate, one would expect a 2/3 power-law dependence, however, due to the complexity of the cells, the spheroids density is not uniform (Fig. 2B). Interestingly, even when the number of cells per spheroid was increased, the area of the spheroid did not grow proportionally, producing more dense spheroids. This phenomena was observed in all cell types (Fig. 2).

### Histological analysis of hybrid spheroids reveals capillary-like structures

**Figure 3:**
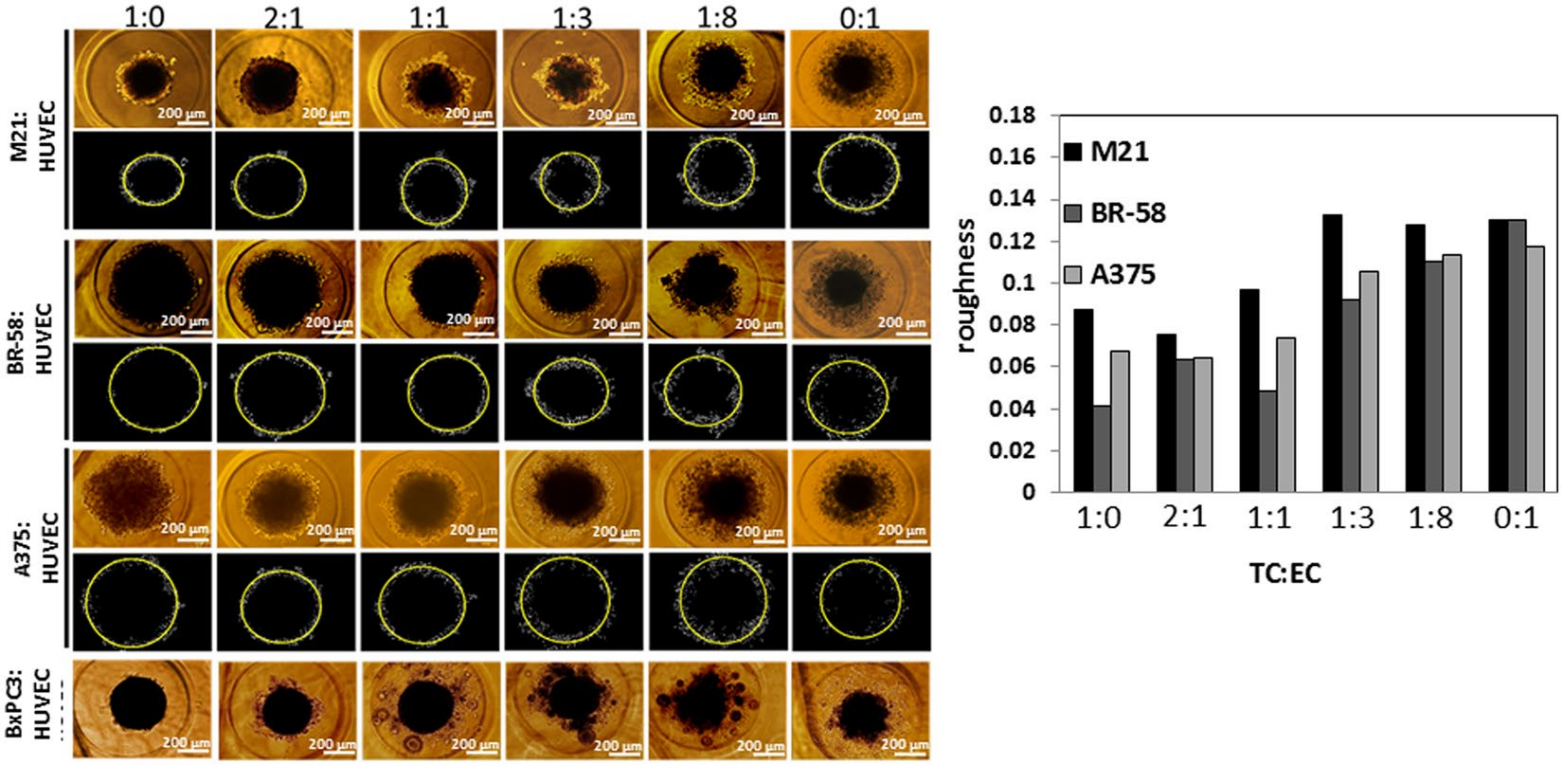
Tumor/Endothelial cell hybrid spheroids. Spheroids’ surface roughness (χ) values were calculated for spheroids composed of M21, BR-58 or A375 with different content of HUVEC cells. Typical images of hybrid spheroids and their corresponding MATLAB image analysis for roughness calculation. In the upper panels are the original images, and under each panel are the spheroid borders (white) with a fitting circle (yellow). BxPC3 spheroids were not analyzed due to satellite spheroid formation. Bar = 100 µm.

**Figure 4:**
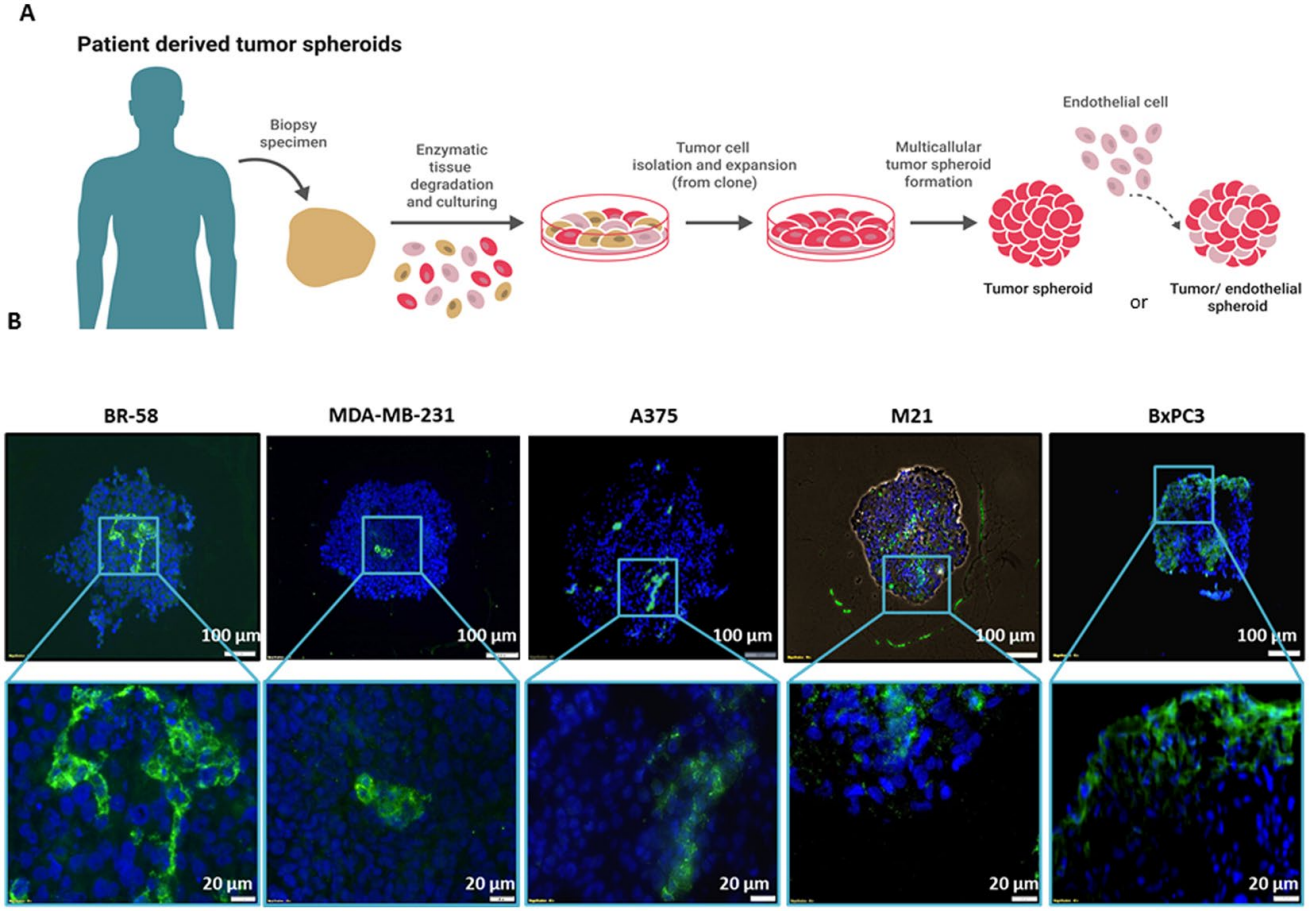
Patient derived spheroids formation process and histological examination of TC:EC hybrid spheroids. **(A)** Illustration showing the isolation of tumor cells from a patient surgical sample, and assembly of tumor spheroids or TC:EC hybrid spheroids. **(B)**Immunofluorescent staining of EC using anti-CD31 in spheroid frozen sections of BR-58, M21, BxPC3 and A375 mixed with HUVEC at a 1:1 ratio. Green = CD31; Blue = nuclei DAPI staining.

To identify capillary-like structures in endothelial TC hybrid spheroids, spheroids (1:1 ratio of TC:EC) were sectioned and stained for CD31 detection of EC, as presented in Fig. 4B. In all analyzed spheroids; BR-58, MDA-MB-231 and M21, EC were organized in clusters, while in BxPC3 spheroids endothelium tended to aggregate in the periphery rather than distribute throughout the micro-tissue. However, in the patient-derived cells, BR-58 and M21, and in A375 cell line, clear capillary-like structures were detected. M21 and BR-58 presented endothelium clustering of cells when DAB staining was coupled with anti-CD31 staining (Fig. 4B, Fig. 6, Fig. 3S). Interestingly, M21 presented an open lumen-shaped capillary-like structure ﻿(Fig. 3S)﻿.

### Interactions between tumor cells and endothelial cells in monolayer co-culture

Co-culture of cells in monolayers were applied in order to study the interactions between specific TC and EC. TC of either BxPC3 or A375 were seeded with HUVEC cells at a 1:1 ratio, with increasing total number of cells at the same ratio. While HUVEC tended to adhere to the surface and spread-out, BxPC3 created separated aggregates, creating only minimal interactions with HUVEC cells. In contrast, A375 presented a clear interaction with HUVEC cells (Fig. 2S), creating a monolayer combined of both TC and EC.

### Higher content of endothelial-cell- in tumor spheroids reduces their surface smoothness

**Figure 5:**
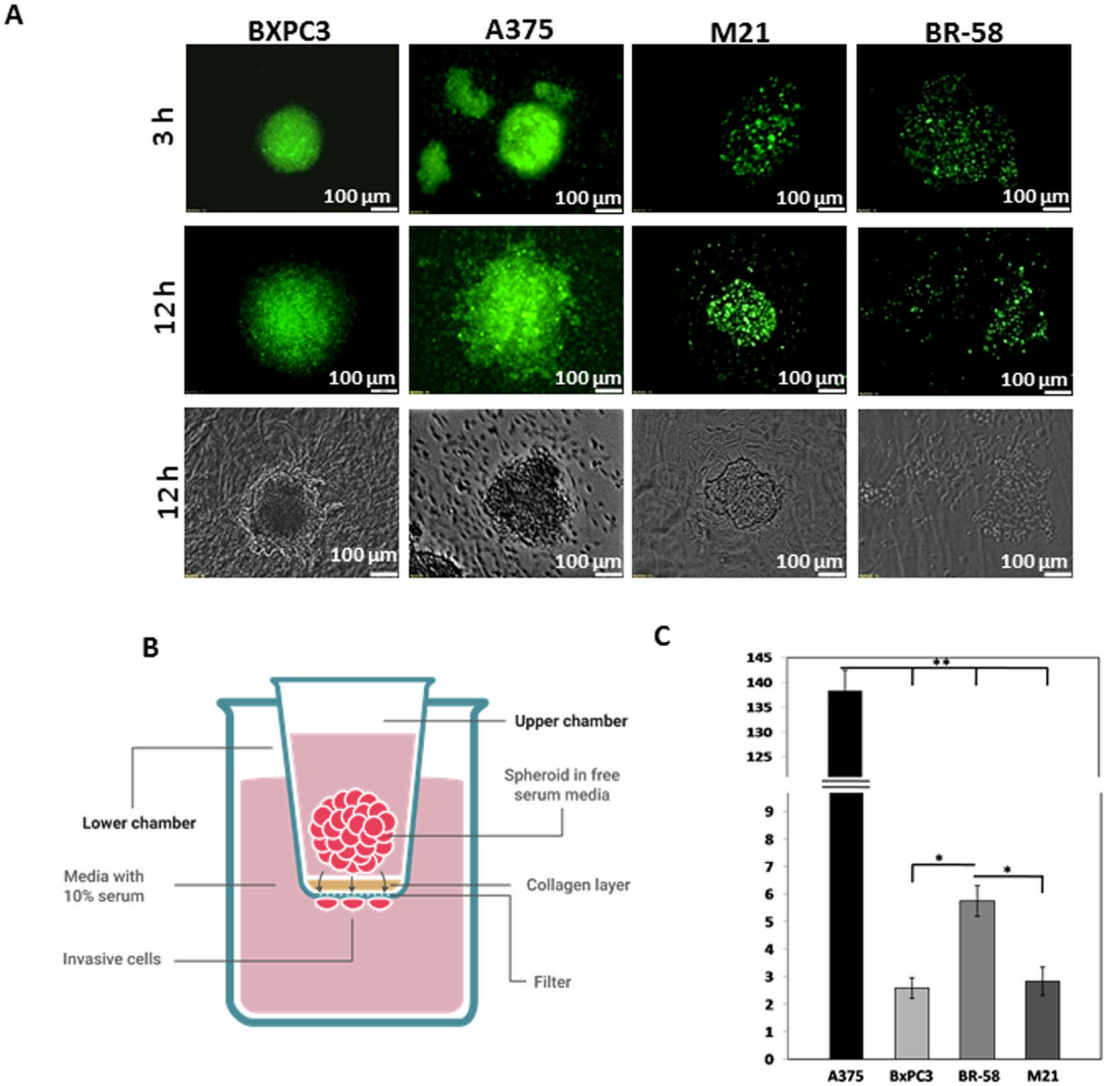
Spatial spheroid invasion assays: **(A)** 3D cultured spheroid made of BxPC3, A375, M21 and BR-58 embedded in collagen type I after 3 and 12 hours (bar = 100 µm). **(B) **Illustration of transwell invasion assay. In this assay, spheroids are seeded onto collagen coated polycarbonate membrane. The upper chamber contains serum free media and the lower chamber is supplemented with 10% serum. After 48 hour invasive cells that ﻿have detached form the spheroids are stained and counted. **(C)** Quantification of cell invasion from spheroids of BxPC3, A375, M21 and BR-58 in transwell assay. n = 12, **p < 0.025, *p < 0.05.

For the purpose of studying the interactions of EC and TC that affect vascularity and capillary-like structures, we grew spheroids in different TC:EC ratios and measured their overall morphology, size, surface and outline. Figure 3 shows typical spheroids made of four cell types: M21, BR-58, A375 and BxPC3, with a total number of 10,000 cells per spheroid. As seen in Fig. 3, the change in morphology was consistent with the increase of EC content and with a rougher surface texture. Bright field images of BxPC3 show that this specific cell type formed the smallest and most dense spheroids, compared with all other cell types. To parameterize the smoothness of the spheroids’ surface, we performed an image analysis using MATLAB for representative images of the spheroids array. We defined χ as the roughness of a spheroid, accounting for the local dispersity of the border coordinates; the smaller χ value is- the smoother the spheroid surface is. As demonstrated by M21, BR-58 and A375 (Fig. 3), increased number of HUVEC cells, roughens the margins of the spheroids as reflected by higher χ values. Interestingly, χ value seems to correlate with the invasiveness potential of the spheroids (Fig. 5). Intuitively, the rougher the spheroid is, the more cells  detach from its margin and the higher invasive potential it has. This analysis is only a qualitative evaluation based on a single representative image per spheroid’s TC:EC ratio. Unfortunately, ﻿as a result of BxPC3 spheroids’ structure, with high dispersity and many small “semi-spheroids” around them, we were unable to resolve the structures’ smoothness and roundness (Fig. 3).

### Tumor origin intrinsically affects spheroids’ cells invasiveness

To measure the invasiveness level of TC from spheroids, two experiments were performed in fluorescently-labeled A375, M21, BR-58 and BxPC3 spheroids. In the first experiment, spheroids were seeded onto a layer of collagen type I and images were taken using a fluorescent microscope after 3 and 12 hours, to monitor their progress of invading the gel. After 3 hours, A375 cells had invaded the gel compartment, M21 and BR-58 presented no invasive traits at that time point, and BxPC3 showed only sporadic migration in the vicinity of the spheroid’s surface. After 12 hours substantial invasiveness was detected in all cell types (Fig. 5A). A375 and BR-58 were both more invasive than BxPC3 and M21.

The second assay was a quantified invasion assay in which spheroids were grown in U-bottom shaped 96-well plates. The spheroids were then seeded in 96-transwell plates, coated with collagen type I and the number of invading cells was detected after 48 hours. As illustrated in Fig. 5B, cells that permeated the membrane and reached the distant side of it, were stained and counted. The number of invading A375 cells was significantly higher than that of other cell types, with A375 presenting to be approximately 27 times more invasive than BR-58 (Fig. 5C), and M21 45-fold less invasive than A375. BR-58 spheroids were about twice more invasive than M21 and BxPC3.

### Patient-derived tumor spheroids produce high levels of collagen

**Figure 6:**
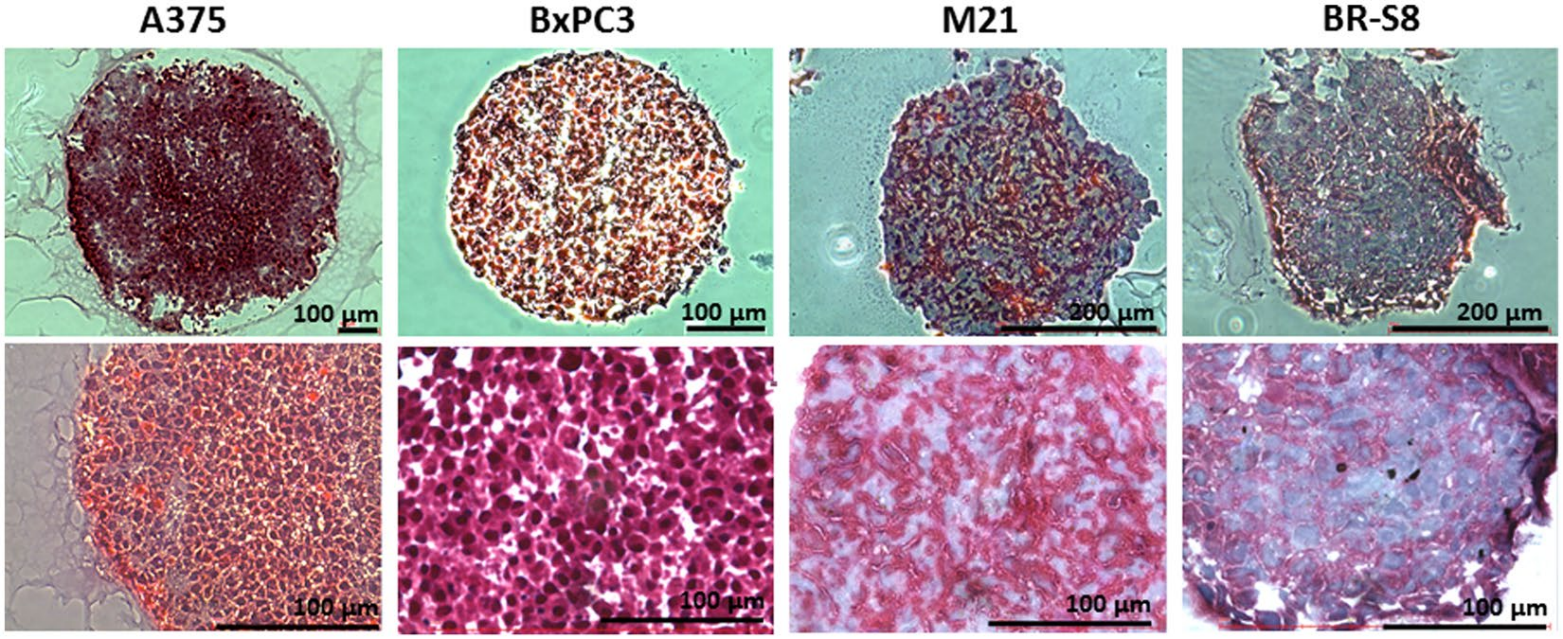
Histological staining for Collagen level in spheroid sections. Masson’s trichrome staining as performed in frozen sections of BR-58, M21, BxPC3 and A375 spheroids. Collagen is stained in blue. Bar = 100 µm.

Spheroids composed of either BxPC3, A375, M21 or BR-58 cells were fabricated using the 3D Petri dish method, fixed and sectioned for histological analysis of their ECM content. The spheroid sections were stained by Masson's staining for collagen detection. As presented in Fig. 6, BR-58 and M21,﻿﻿ primary cells originating from cancer patients, generated higher levels of ECM than that produced by BxPC3 and A375 cancer cell lines, as seen by their weaker blue staining.

## Discussion

Despite successful pre-clinical testing, 85% of early clinical trials for novel drugs fail, and of those that continue to phase III, only 50% pass all the required approvals for clinical use^39^. Out of all drug trials, oncology drug trials suffer from the highest rate of failures^40^.

The accelerated advances in tissue engineering and microfabrication techniques, have dramatically improved the ability to grow and maintain cell cultures, tissue fragments and organoids *ex vivo*. 3D cell models, which can simulate the tumor microenvironment, could substantially improve the validity of the assays, and better predict drugs’ success^41–44^.

Previous studies show that cell behavior is affected by the presence of stromal cells, either alone^45^ or in combination with mesenchymal cells^11^, which exist in the tumor microenvironment. For instance, different invasion patterns and cytotoxic properties were observed when monitoring the drug effect on fibroblasts alone compared with co-culture of varying ratios of fibroblasts and cancer cells^46, 47^. Interactions with inflammatory cells, such as macrophages at the spheroids’ surface were also studied, showing their ability to infiltrate the inner layers of these spheroids, and disintegrate them after 5 days^48, 49^.

Therefore, in this study, we investigated the interactions between EC, representing tumor vasculature as a major micro-environmental component, and TC. After comparing four different methods for spheroid formation (Fig. 1), we found that the 3D Petri dish array had formed the most robust and standard spheroids; homogenous in both size and circular shape. However, in some cases the spheroids’ size was physically limited by that of the well, which may have an effect on cellular processes such as proliferation and apoptosis^50–52^. Compared with the 3D Petri dish array method, agarose-coated flat wells yielded heterogeneous spheroids and in many cases there were multiple spheroids per well. Formation of spheroids with the Lipidure^®^ MPC-coated 96-well plates yielded better results than those of the agarose method, with single spheroid per well and spheroids of considerably same round shape. Such results may be attributed to the U-shaped bottom of these plates that prevents the cells from spreading over the bottom and forces them to self-interact, together with the MPC coating that prevents cells from adhering to the surface of the plate. Despite their rounded shape, spheroids were quite polydispersed, as demonstrated in Fig. 1. The hanging-drop method, which is a commonly used technique for spheroid formation, created a wide range of spheroids, both in terms of size and shape, in most cells types. Similar to our findings, in a previously published work performed with ovarian cancer cell lines, the hanging-drop method did not seem to yield very round spheroids^53^, though a different study showed to produce rather round spheroids using OVCAR8 cell line. Additionally, this method requires specific optimization per cell type, since the time required for formation is different for each cell type. All three methods; hanging-drop assay, U-shaped bottom coated 96-well plate and agarose-coated plates, formed spheroids of varying sizes and shapes and it appears that each cell type requires specific optimization. Another method used for scaffold free spheroid formation is the Perfecta3D^®^ Hanging Drop Plate from 3D Biomatrix™, which is basically a standardization of the hanging drop method using designated plates^54^. The downsides of this method, is the requirement of an additional step for spheroid formation, and the cost of the special designated plates which are non-reusable.

However, the 3D Petri dish^®^ array allows for better size and shape control of the spheroids, regardless the origin of cells. The special wells that are created with the re-usable mold, physically restrict the spheroids of further growth and force a same size spheroid formation (Fig. 1S).

Using the Petri dish^®^ array, we compared aggregates of two patient derived cell types; M21 and BR-58, and two cancer cell lines of different origin; A375 and BxPC3, using different cell number per spheroid. In all four cell types, spheroids’ density increased with the elevation of cell number per spheroid. BxPC3, a pancreatic cancer cell line, presented round spheroids even when density was increased, as seen in Fig. 2A. This observation is consistent with the known pathophysiology of pancreatic tumors, that present with fibro inflammation, dense stromal tissue fibrosis and hypo-vascularity. In fact, not only are these tumors poorly vascularized, but the blood vessels within the tissue are also commonly compressed as a result of the high tissue pressure^55^. These tissue characteristics have a substantial effect on drug permeability into the tumor via diffusion^56^. Our observations suggest that the spheroid’s shape and condensed structure may be signs of cluster growth.

A functional endothelium is extremely vital for the tumor’s growth, and the structural patterns of the endothelium in the tissue seem to indicate tumor aggressiveness and disease prognosis. In fact, extravascular migratory metastasis in melanoma and angiotropism, “pericytic mimicry” for instance, or the replacement of pericytes by angiotropic TC, are considered to be possible biomarkers for poor prognosis of that type of cancer^57, 58^. This suggests that angiogenesis is not required for these tumors when growing in the vicinity of pre-existing blood vessels, but the interaction with endothelial cells is critical.

Since this spheroid model is static, we do not expect formation of functional capillaries, but we aimed to better understand whether the formation of primitive vessel architecture is tumor-cell-type dependent, and whether capillary structures can be generated without a steady flow. To investigate this, we co-cultured HUVEC with TC using different ratios to form spheroids. We compared different tumor types, “forcing” a fixed TC:EC ratio that may differ than that usually found *in vivo*. Nevertheless, we detected dramatic differences between cell types. For example, immunofluorescence staining revealed that EC in BxPC3 spheroids tend to cluster closer to the spheroids’ periphery rather than to the core, as seen in most cases (Fig. 4). Additionally, their structure did not reassemble into tubular shapes. This is in contrast to BR-58, MDA-MB-231 and A375 spheroids, all originated from highly angiogenic tumors, which correlatively formed a vessel-like network. As expected this vessel-like network was non-functional. Notably, as depicted in Fig. 3S, spontaneously assembled “open lumen”-like structures were detected in M21 spheroids. A similar pattern of CD31 staining, showing endothelial cell organization in the center of the spheroids, was presented in a recently published study done with patient-derived papillary thyroid cancer cells and normal thyroid spheroids cell culture^59^. Interestingly, in another recent study, Lamichhane *et al*. demonstrated the use of a triple co-culture 3D microenvironment recapitulating system. In this study, human MSC were co-cultured with human pulmonary vascular endothelial (HPMEC) and lung epithelium A549 lung cancer cells, to form an *in vivo* like model^11^. The authors elegantly show that the presence of MSC may act as an important factor in the long-term sustainability of viable endothelial cells in hypoxic regions, thus providing a better *in vivo* mimicking model.

To further elucidate whether the spontaneous assembly of capillary like structure is innate, we followed our investigation using the 2D monolayer growth model, to explore interactions between cells. When we co-cultured EC and TC at a 1:1 ratio (Fig. 2S), we found that under confluent conditions TC form islands of aggregates that are surrounded by a flat monolayer of EC. Remarkably, in this 2D set up, when comparing BxPC3 with A375, the island growth pattern presented higher expression in the BxPC3 pancreatic cell line, which correlates with the tendency of the cells to create distinct aggregates in the 3D spheroids.

Cell invasiveness is a critical key function in the metastatic cascade. Our observations indicate that with higher tumor HUVEC content, spheroids produce rougher margins as more cells “sprout” or detach from the central aggregate, suggesting higher invasiveness potential (Fig. 3). We were interested to learn whether the innate potential of TC to invade the ECM can be maintained in a 3D *ex vivo* structure, and whether we can detect significant differences in the invasiveness of spheroids based on their tumor type. We found that each cell type has different migratory potential that is not preserved across all tumor types. For example, A375 is highly invasive in collagen type I, whereas M21, a patient-derived cell type originating from the metastatic site of the lymph node, was found to be 45-fold less invasive than A375. This suggests that cancer cells’ behavior substantially differ in cell lines comparing with primary TC, therefore may not be used as a reliable tool for predicting clinical behaviors.

We found that spheroids of M21 and BR-58, both patient derived tumor spheroids, produced high level of collagen as seen in Fig. 6. Considering the effect of ECM collagen deposition on both tumor volume^60^ and progression^61^, further studies are needed to sample a larger number of spheroids of different origins in order to shed light on such possible correlations.

Our findings conclude that the 3D model of hybrid spheroids may be successfully constructed, but since each tumor type generates different interactions with its neighbor cells and EC, there is a need of a platform for personalized medicine that would account for these differences. Notably, we found that the hybrid spheroid of EC and TC can present vascular-like structures that may sprout when interacting with ECM. Such a model would provide the basis for complex multicellular tumors and stromal structures, to be used as a more reliable prediction method of biological processes and drug efficacy.

## Electronic supplementary material

Supplementary 1
