# Supplementary material for: Tumor cells and their crosstalk with endothelial cells in 3D spheroids
Source: Sci Rep. 2017 Sep 5;7:10428. doi: 10.1038/s41598-017-10699-y (PMC5585367; doi:10.1038/s41598-017-10699-y)
Supplement: Supplementary file 1 — Supplementary 1 [file 41598_2017_10699_MOESM1_ESM.pdf]

Tumor cells and their crosstalk with endothelial cells in 3D spheroids

Hila Shoval[1], Adi Karsch-Bluman[1*], Yifat Brill-Karniely[1*], Tal Stern [1], Gideon Zamir[2], Ayala Hubert[3], Ofra Benny[1]

1. The Institute for Drug Research, The School of Pharmacy, Faculty of Medicine, The Hebrew University, Jerusalem, Israel
2. Department of Surgery and Transplantation Unit, Hadassah Medical Center, Ein-Karem Jerusalem, Israel.
3. Oncology Department- Hadassah Medical Center, Ein-Karem, Jerusalem, Israel

* Equally contributed

Corresponding Author: Ofra Benny, Institute for Drug Research, The School of Pharmacy, Faculty of Medicine, Campus Ein Karem, The Hebrew University, Jerusalem 91120, Israel. Phone: 972-2-6757268; Fax 972-2-6757273; Email: ofrab@ekmd.huji.ac.il

Supplementary method

Methods

2D cell co-culture

Cells were seeded in duplicates in 6-well plates in ratios of 1:1 TC: EC in different densities of A375 or BxPC3 mixed with HUVEC (TC: EC 100,000, 200,000 and 400,000, as control duplicates of 300,000 HUVEC, 300,000 BxPC3 and 200,000 A375 cells were seeded). Each of the cell type, HUVEC, BxPC3 and A375 cells were cultured individually. Control plates of BxPC3 and HUVEC were seeded with 3 X 10^5 cells, and A375 with 3 X 10^5 due to their tendency of rapid growth. Cell cultures were monitored and pictures were taken after 12 hours using a light microscope (Olympus IX73).

Immunohistochemistry analysis

For histochemical staining, sections were fixed in PFA 4% for 20 min and washed 3 times for 5 min. 3% hydrogen peroxide in methanol solution was used to quench peroxidase activity. Sections were incubated for 30 min in 2.5% normal horse serum (Vector Laboratories, CA, USA) for blocking followed by anti-CD31 incubation (Abcam, MA, USA 1:50) in 2.5% normal horse serum added for overnight incubation at 4°C. Slides were washed 3 times with PBS and incubated in IgG Immpress reagent kit secondary antibody (Vector Laboratories, CA, USA) for 30 min at RT. Sections were then washed 3 times and reacted with peroxidase using a DAB substrate kit (Vector Laboratories, CA, USA). The sections were counterstained with Hematoxylin, mounting media was added and covered using a coverslip. For collagen

staining, frozen sections were thawed at RT for 30 min, fixed in 10% formalin for 10 min and washed 3 times with PBS. After 3 washes the sections were stained with Trichrome dye (Connective Tissue Stain) using Masson Trichrome Staining kit according to manufacturer's instructions (Abcam, MA, USA).

Figure 1S

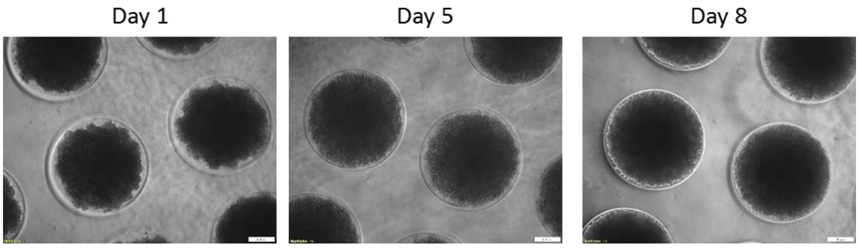

Figure 2S

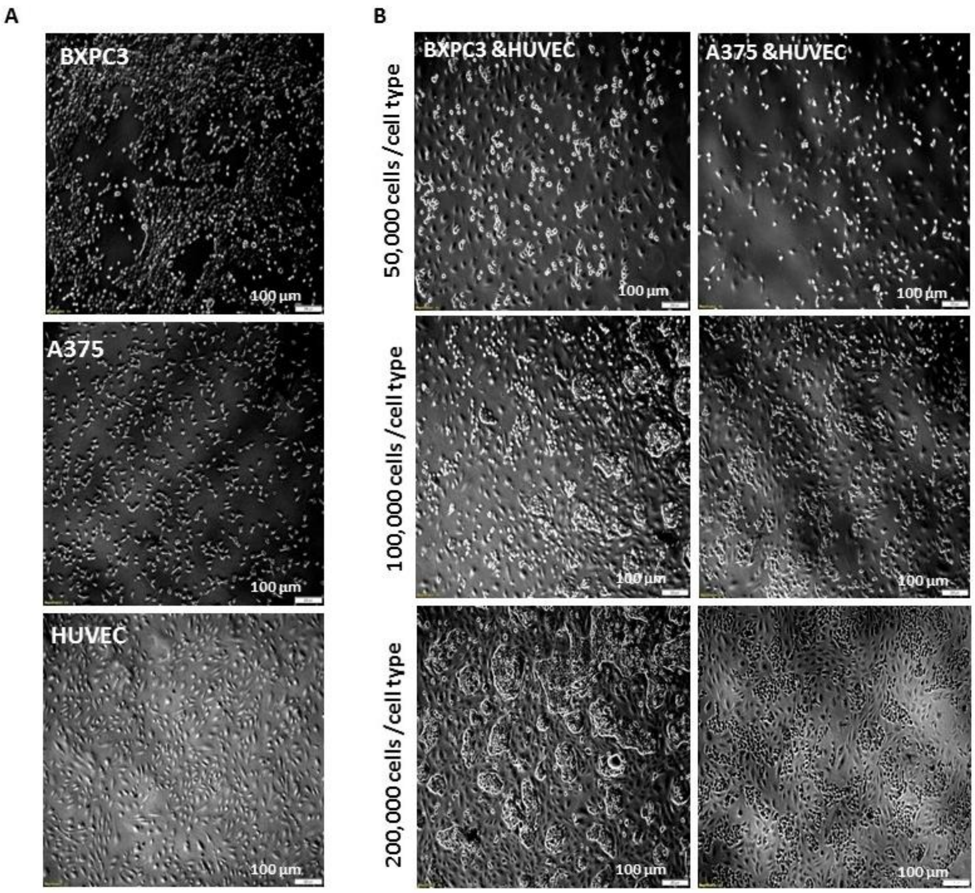

Figure 3S

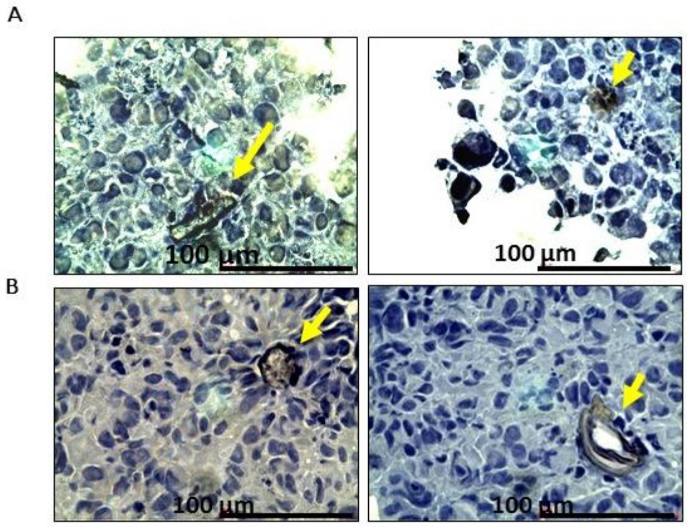

Figure legend

Figure 1S. A375 spheroids cultured in 3D-Petri-dish method. Pictures were taken every day. Representative pictures of the same size of spheroids over time.

Figure 2S. Tumor and endothelial cell co-culture monolayers. **(A)** Cell culture of single cell type of the following cells: HUVEC, BxPC3 and A375 **(B)** Co-culture of 1:1 TC:EC of HUVEC cells with either BxPC3 or A375 at different cell number. Bar= 100 µm.

Figure 3S. Hybrid **s**pheroids composed of patient-derived tumor cells and HUVECs (1:1) immunostained with CD31 followed by DAB histochemical staining of frozen sections of **(A)** BR-58 and **(B)** M21 spheroids. Brown = CD31; Blue/Purple= nuclei, hematoxylin staining. Bar= 100 µm. Arrows indicate sites of vessel-like structures, lower right image of M21 spheroid showing “open lumen” like structure.

Table legend

Table 1S. A table concluding all the cells features of the cell lines used in this study.

Table 1S

**TC**	**Origin**	**Features**
**PANC1**	Human pancreas/duct epithelioid carcinoma	ATCC® cell line
**MDA-MB-231**	Human adenocarcinoma mammary gland/breast; derived from metastatic site	ATCC® cell line
**BxPC3**	Human pancreas Adenocarcinoma	ATCC® cell line
**A375**	Human skin malignant melanoma	ATCC® cell line
**BR-58**	Human primary breast cancer derived mammary tumor cells. HER-positive mammary tumor cells	Patient derived tumor cells
**M21**	Human primary melanoma cells from lymph node	Patient derived tumor cells
**HUVEC**	Human umbilical vein vascular endothelium	Lonza® endothelial cells
